# Associations of work characteristics with obesity, behavioral risk factors and NCDs in Bangkok, Thailand

**DOI:** 10.1371/journal.pgph.0004000

**Published:** 2024-12-05

**Authors:** Sopit Nasueb, Hathairat Kosiyaporn, Nisachol Cetthakrikul, Rujira Adhibai, Jiranun Thiphong, Yanisa Pumsutas, Orratai Waleewong

**Affiliations:** International Health Policy Program, Ministry of Public Health, Nonthaburi, Thailand; Tribhuvan University Institute of Medicine, NEPAL

## Abstract

Non-communicable diseases (NCDs) are one of the premature causes of death in Thailand especially among working age group. This study aims to examine the associations between work characteristics and NCDs, obesity, and behavioral risk factors in Bangkok, the Capital of Thailand. This study employed secondary data analysis of cross-sectional data from the Health Behavior of Population Survey 2021 by the National Statistical Office (NSO). The respondents who were 15–60 years old and resided in Bangkok were included in this study (n = 4,925). The data were analyzed by descriptive statistics of work characteristics, and multiple logistic regression between working groups and behavioral risks adjusted with other demographic and socioeconomic variables. The study found that all workers showed a lower likelihood of reporting NCDs compared to the unemployed. Professional/administrative/managerial workers had 44% less chances of reporting NCDs compared to unemployed (AOR = 0.56; 95%CI = 0.43–0.75; P-value <0.001. All working groups showed a significant association with alcohol consumption, smoking, and dietary intake. In particular, skilled, semi-skilled, unskilled workers, technicians, clerks, and service or sales workers were more likely to smoke and drink alcohol compared to those who were unemployed. Nevertheless, work characteristics did not affect the likelihood of inappropriate fruit and vegetable intake, insufficient physical activity, and sedentary behavior. The study found a link between work characteristics and NCDs in the working-age population, identifying specific work characteristics associated with behavioral risk factors such as alcohol consumption, smoking, and high dietary risk. The findings suggest a need for NCD prevention strategies targeting diverse workplaces such as smoking regulations, healthy canteens, and promoting opportunities for physical activity, with a regulatory focus on labor laws and policy incentives. Finally, disaggregated occupational data should be emphasis for effective monitoring and evaluation in NCD policy.

## Introduction

Premature deaths due to non-communicable diseases (NCDs) in Thailand constituted over 72% of the total mortalities, with stroke and diabetes mellitus as the two leading causes of NCD-related deaths [1,2]. Moreover, in 2009, the economic cost of NCDs was approximately USD 6,617 million due to premature deaths and loss of productivity among the workforce [3]. The underlying causes of behavioral risk factors of NCDs include tobacco use, insufficient physical activity, unhealthy diets, and harmful use of alcohol [3] The recent survey, the National Health Behavior Survey (NHBS) discovered 29.5% of Thai people aged 15 years and over were current smokers, 44.6% were current drinkers, and 30.9% reported having insufficient physical activity [4]. Additionally, most working people did not eat three meals per day and consumed less than the standard proportion of fruits and vegetables [4]. Therefore, NCDs and their behavioral risks should be focused on the working age group that mainly contributes to economics.

Social determinants of health and social-environmental factors such as neighborhoods in which people live and work and socioeconomic conditions such as income and occupation affect individual health and are linked to the escalation of NCDs [5–7]. Among the working population (15–59 years old), work characteristics and workplace risk factors and hazards, such as sedentary work, work stress, exposure to carcinogens, shift work, and unsupportive environments, can cause, or exacerbate NCDs and lead to increasing in mortality [8]. These factors contribute to disparities in exposure to NCD risk factors, accessibility to health resources, and health outcomes [9]. Health disparities seem to be prominent in an urban area or a capital city, where rapid growth due to national economic development took effect [8]. Accordingly, employment or occupation is one of the socioeconomic indicators for tracking these health disparities among people in communities.

Like other capital cities, Bangkok, Thailand’s most urbanized area, has experienced rapid urbanization, leading to changes in lifestyle and behavior, including decreased physical activity, increased consumption of processed foods, and exposure to environmental pollutants [10,11]. In Bangkok has a working-age population (aged 15 years and older) of 7,868,190 people, where over five million people are employed [12]. The majority are workers, contributing to a higher risk of NCDs especially obesity, diabetes, and hypertension within the working population [13]. A study on diabetes among office workers in Bangkok found that the incidence rates were 11.8 per 1000 person-years for ages 35–39, 6.8 for ages 40–44, and the highest rate of 23.6 for ages 55–59 [14]. To deal with the complexity of NCD prevention, occupational status in Bangkok needs to be considered.

Many comparative studies have examined health status and occupational factors that influence health outcomes, but it is often among certain groups such as immigrant workers and informal workers with more focus on access to social protection and mostly conducted in other countries [15–17]. It is little known about the occurrence of NCDs among the general workforce and their associations with occupational or working conditions as health inequalities in Thailand. Understanding the relationship between occupational classification and NCDs, including behavior risk factors can provide valuable information for developing NCD prevention and control measures to reduce the burden of NCDs among workers in different work settings as a surveillance system [18]. According to the diversity in occupational characteristics in Bangkok, this study aims to assess the association between work characteristics, with NCDs, obesity and behavioral risk factors in this area.

## Material and methods

### Study design and data source

**Fig 1:**
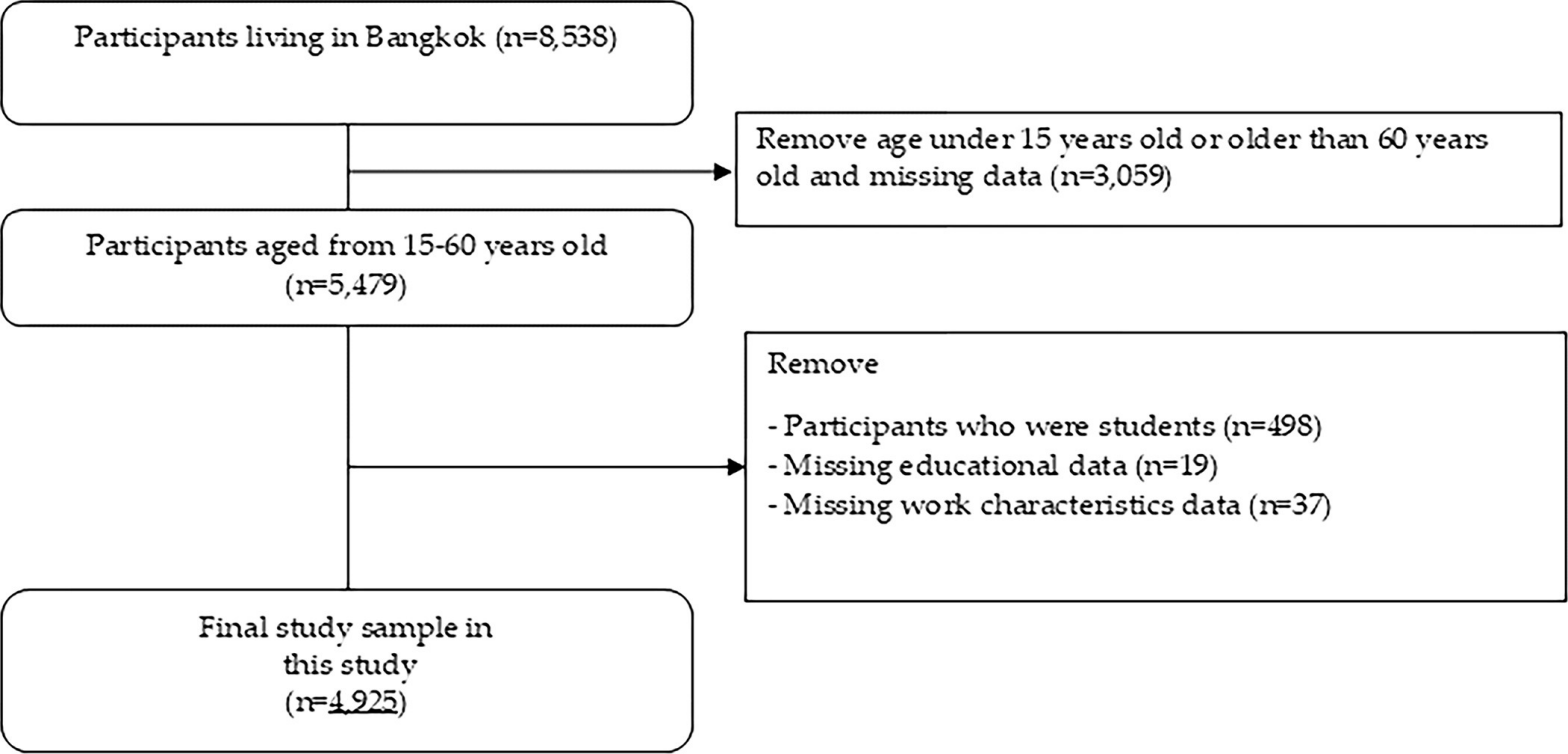
Study sample flow chart.

This study applied secondary data analysis from the National Health Behavior Survey (NHBS) 2021, a cross-sectional survey with national representative samples, conducted by the National Statistical Office (NSO) [4]. The two-stage cluster survey was representative of the Thai population with clusters of both municipal and non-municipal areas as the primary sample and households as secondary samples, resulting in a total of 84,000 households (n = 207,191). This survey was undertaken from February 1^st^ to May 31^st^, 2021. However, only respondents aged 15 to 60 years and residing in Bangkok were included in the analysis of work characteristics (n = 4,925) (Fig 1), as this age range is legally defined as the working age in Thailand [19,20].

### Data measurement

The data for this study were drawn from the 2021 Health Behavior of Population Survey, which utilized a secondary dataset of Thai nationals. A two-stage stratified sampling method was applied across 77 provinces in Thailand. Provinces were used as the primary strata, with each further divided into municipal and non-municipal sub-strata. Within each sub-stratum, enumeration areas (EAs) were randomly selected, followed by a random selection of households, yielding a sample of 84,000 households. All members of these households were invited to participate in the survey, which was conducted by NSO. A total of 207,191 participants were involved. Participants were informed through official letters sent to community leaders and individuals before the face-to-face interviews, which were conducted nationwide between February 1 and May 31, 2021. Interviews were conducted in Thai and later translated into English for the purposes of this article. Data was adjusted for non-response before handling to researcher. The summarized results, full report, and downloadable Excel files can be accessed at the NSO website (https://www.nso.go.th/nsoweb/nso/survey_detail/w6?set_lang=en).

Seven measurements of the NHBS 2021 were included in this study [4]. Independent variable of work characteristics was derived from occupation question items. They were reclassified based on the European Socio-economic Classification (EseC) to reflect social classes in a concept of employment relations [21]. Five work characteristics in this study were classified as (i) unemployed people (including housework, looking for work or unwilling to work), (ii) professional, administrative, or managerial workers (including doctors, chief executive officers, and managers), (iii) technicians or clerks, (iv) service or sale workers, and (v) skilled, semi-skilled, or unskilled workers (including labors, craftsmen, cleaners). Groups ii and iii were considered as higher-skill roles in society than groups iv and v.

In addition, demographics and socioeconomic characteristics were covariate factors. It comprised of gender (classified as male or female), age group (classified as 15–24, 25–44, and 45–60 years old), and education level (classified as illiterate, primary school, secondary school, and diploma or higher). Wealth index quintiles of total samples calculated from household characteristics and assets, in which the first quintile referred to the poorest group whereas the fifth quintile was the richest group.

Dependent variables were NCDs, obesity, and behavioral risk factors. Five self-reported diagnosis of NCDs were selected in the analyses including hypertension (HT), diabetes mellitus (DM), dyslipidemia (DLP), cardiovascular disease (CVD) and chronic obstructive pulmonary disease (COPD) classified as having NCDs or healthy groups. The self-reported body weights and heights were used to calculate Body Mass Index (BMI). Individuals with a BMI of 23–24.5 kg/m were identified as overweight, while those with a BMI over 25 kg/m were classified as obese, following the Asian BMI cut-off [22]. However, in this study, we treated overweight and obesity as a single variable classified as appropriate BMI and overweight/obese groups.

Six NCD behavioral risk factors were (i) tobacco use (non-smoker vs current smoker who smoked in the past 12 months), (ii) alcohol consumption (abstainer vs current drinker who drank in the past 12 months), (iii) high dietary risks (low vs high dietary risk who reported more frequent consumption of salty food, high in fat food, and sugar-sweetened beverages (SSB) every day or at least 5–6 days per week), (iv) inappropriate fruit and vegetable intake (insufficient or sufficient intake who consumed every day or at least 5–6 days per week), (v) physical activity (sufficient physical activity vs insufficient physical activity) and (vi) sedentary behaviors (sedentary vs non-sedentary behaviors). Physical activity outcomes drawn from responses of a modified Global Physical Activity Questionnaire (GPAQ) developed by the World Health Organization (WHO), calculated to metabolic equivalent of task (MET-min per week or minutes consumed in each physical activity level per week), and classified as “insufficient” or “sufficient (600 MET/minute/week”) [23]. Sedentary behaviors are defined based on numbers of hours of sitting or reclining per day (“sedentary” or “non-sedentary, ≥ 7 hours per day) [24].

Ethical review and approval were not required for this study, as it utilized data from the Health Behavior of Population Survey 2021. This dataset is part of the national routine household surveys conducted by the National Statistical Office (NSO), a government agency. According to the Statistics Act of 2007 (refer to https://www.nso.go.th/nsoweb/storage/contents_detail/2023/20230520172939_25785.pdf), the NSO is not obligated to obtain signed consent forms from respondents. However, Article 15 of the Act ensures the confidentiality of data collected by NSO fieldworkers and other users of the dataset. In compliance with this legal requirement, the researchers in this study strictly adhered to Article 15, maintaining the confidentiality of all individual information, which was not disclosed or reported in the publication.

### Data analysis

To address missing data in this article, individuals with incomplete information on key independent variables related to work characteristics (n = 37) and covariates (n = 19) were excluded. Furthermore, participants who fell outside the study’s scope including those over the age of 60, those under 15, and those identified as students—were also excluded from the analysis (see Fig 1).

Statistical analyses were performed in STATA, version 17.0 (Serial number: 401709350741). This study applied two statistical analysis techniques. First, descriptive analysis was used to explore the frequency and percentage of demographic characteristics of each work characteristic group; and NCDs, overweight/obesity and behavioral risk factors of each work characteristic group. Second, we applied multiple logistic regression to analyze the association between work characteristic variables, NCDs and overweight/obesity, as well as a relationship with behavioral risk factors. We employed the Hosmer-Lemeshow goodness-of-fit test to assess the adequacy of model fit across all statistical models. It is indicated by *p*-value, which should be >0.05 to be fit. Other demographic and socioeconomic variables were confounding factors in the multivariate analysis. Level of significance was set at 5% or *p*-value less than 0.05 was defined as statistically significance.

## Results

### Demographic characteristics by work characteristics

Among samples (N = 4,925), 29.6% were skilled, semi-skilled or unskilled workers, followed by professional, administrative, or managerial workers (22.3%), service or sale workers (19.2%), technicians or clerks (14.5%) and unemployed people (14.4%). The majority of all work characteristics were female (55.6–72.1%) while skilled, semi-skilled, and unskilled workers were mostly male (63.7%). Most of the unemployed samples were in the 45–60 years age group (56.3%), whereas other work characteristics groups were predominantly in the 25–44 years age group (47.4–61.0%). For educational level, service or sale workers (45.8%), and skilled or semi-skilled or unskilled workers (45.3%) mostly graduated from a secondary school, while professional or administrative or managerial workers (77.9%), technicians or clerks (59.2%), and unemployed people (36.0%) received a diploma or higher degree. The large number of unemployed people (35.6 and 36.5%), technicians or clerks (36.5 and 36.9%), service or sale workers (26.9 and 24.5%), and professional or administrative or managerial workers (44.3 and 43.9%) were in the richest groups (quintiles 4 and 5), while most of skilled or semi-skilled or unskilled workers was in the poorest group (quintile 1) (30.0%) (Table 1).

**Table 1:** Demographic characteristics, health status and behavioral risk factors by work characteristics.

	Work characteristics, n (%)						Total samples (n = 4,925)
	Unemployed people (N = 710)	Professional/ Administrative/Managerial workers (N = 1,097)	Technicians/ Clerks (N = 716)	Service/Sale workers (N = 946)	Skilled/ Semi-Skilled/ Unskilled Workers (N = 1,456)	X^2^	
**Demographic characteristics**							
Gender						<0.001	4,925
Female	512 (72.1)	638 (58.2)	398 (55.6)	542 (57.3)	529 (36.3)		2,619 (53.2)
Male	198 (27.9)	459 (41.8)	318 (44.4)	404 (42.7)	927 (63.7)		2,306 (46.8)
**Age group**						<0.001	4,925
15–24 years	75 (10.6)	47 (4.3)	51 (7.1)	96 (10.2)	147 (10.1)		416 (8.4)
25–44 years	235 (33.1)	595 (54.2)	437 (61.0)	479 (50.6)	690 (47.4)		2,436 (49.5)
45–60 years	400 (56.3)	455 (41.5)	228 (31.8)	371 (39.2)	619 (42.5)		2,073 (42.1)
**Education**						<0.001	4,925
Illiterate	19 (2.7)	5 (0.5)	6 (0.8)	21 (2.2)	69 (4.7)		120 (2.4)
Primary	200 (28.2)	86 (7.8)	68 (9.5)	271 (28.7)	586 (40.3)		1,211 (24.6)
Secondary	235 (33.1)	152 (13.9)	218 (30.5)	433 (45.8)	659 (45.3)		1,697 (34.5)
Diploma or higher	256 (36.0)	854 (77.9)	424 (59.2)	221 (23.4)	142 (9.8)		1,897 (38.2)
**Socio-economic (wealth index)**						<0.001	4,925
Quintile 1	78 (11.0)	29 (2.6)	90 (12.6)	229 (24.2)	437 (30.0)		863 (17.5)
Quintile 2	66 (9.3)	48 (4.4)	50 (7.0)	127 (13.4)	228 (15.7)		519 (10.5)
Quintile 3	54 (7.6)	52 (4.7)	51 (7.1)	104 (11.0)	177 (12.2)		438 (8.9)
Quintile 4	253 (35.6)	486 (44.3)	261 (36.5)	254 (26.9)	323 (22.2)		1,577 (32.0)
Quintile 5	259 (36.5)	482 (43.9)	264 (36.9)	232 (24.5)	291 (20.0)		1,528 (31.0)
**Health status** *							
**Having NCDs**						<0.001	4,767
No	532 (76.7)	948 (88.4)	610 (88.7)	783 (86.7)	1,193 (84.7)		4,066 (85.3)
Yes	162 (23.3)	125 (11.7)	78 (11.3)	120 (13.3)	216 (15.3)		701 (14.7)
**Being overweight/obese**						0.008	4,241
No	293 (48.1)	476 (50.4)	318 (52.0)	391 (48.2)	557 (44.1)		2,035 (48.0)
Yes	316 (51.9)	469 (49.6)	294 (48.0)	420 (51.8)	707 (55.9)		2,206 (52.0)
**Behavioral risk factors** **							
**Being current drinkers**						<0.001	4,925
No	622 (87.6)	847 (77.2)	472 (56.9)	651 (68.8)	818 (56.2)		3,410 (69.2)
Yes	88 (12.4)	250 (22.8)	244 (34.1)	295 (31.2)	638 (43.8)		1,515 (30.8)
**Being current smokers**						<0.001	4,820
No	648 (92.6)	966 (89.4)	595 (84.3)	742 (80.2)	974 (69.1)		3,925 (81.4)
Yes	52 (7.4)	114 (10.6)	111 (15.7)	183 (19.8)	435 (30.9)		895 (18.6)
**Having dietary risks**						<0.001	4,874
Low risk	335 (47.5)	361 (33.3)	226 (32.1)	336 (35.9)	484 (33.5)		1,742 (35.7)
High risk	370 (52.5)	723 (66.7)	479 (67.9)	601 (64.1)	959 (66.5)		3,132 (64.3)
**Consuming fruit and vegetable**						<0.001	4,902
Inappropriate	158 (22.3)	240 (22.0)	182 (25.6)	248 (26.4)	467 (32.2)		1,295 (26.4)
Appropriate	550 (77.7)	851 (78.0)	530 (74.4)	693 (73.7)	983 (67.8)		3,607 (73.6)
**Having physical activity**						0.463	2,395
Sufficient	262 (73.8)	343 (68.6)	226 (68.7)	337 (71.4)	527 (71.3)		1,695 (70.8)
Insufficient	93 (26.2)	157 (31.4)	103 (31.3)	135 (28.6)	212 (28.7)		700 (29.2)
**Having sedentary lifestyle**						0.007	2,395
No	65 (18.3)	83 (16.6)	66 (20.1)	106 (22.5)	182 (24.6)		502 (21.0)
Yes	290 (81.7)	417 (83.4)	263 (79.9)	366 (77.5)	557 (75.4)		1,893 (79.0)

**X**^**2**^ = chi-square.

*Health status: Missing NCDs data (n = 158), missing overweight/obese data (n = 684).

**Behavioral risk factors: Missing current smoking data (n = 105), missing dietary risk data (n = 51), missing fruit and vegetable consumption data (n = 23), missing physical activity data (n = 2,530) and missing sedentary lifestyle data (n = 2,530).

### Health status by work characteristics and their associations

*The models reported below confirmed that were fit by Hosmer and Lemeshow goodness of fit test (p-value>0*.*05)*. Among the unemployed, the reported proportion of NCDs was the highest at 23.3%, whereas skilled, semi-skilled, and unskilled workers exhibited the highest proportion of overweight or obesity at 55.9% compared to other work characteristics (Table 1). Based on the multivariate logistic regression analyses, professional/administrative/managerial workers had 44% less chance of reporting NCDs compared to unemployed (AOR = 0.56; 95%CI = 0.43–0.75; *p-*value <0.001). We found similar patterns of associations in other work characteristics groups (skilled, semi-skilled, or unskilled workers with AOR = 0.63, 95%CI = 0.48–0.81, P-value <0.001; service or sale workers with AOR = 0.59, 95%CI = 0.45–0.78, P-value <0.001; and technicians or clerks with AOR = 0.65, 95%CI = 0.48–0.90; P-value = 0.008). For overweight or obese, no significant associations were found between overweight or obese and any of the work characteristics (Table 2).

**Table 2:** Multivariate analysis between work characteristics, and NCDs and obesity*.

Work characteristics	NCDs (n = 4,767)			Overweight or Obese (n = 4,241)		
	AOR	95% CI	P-value	AOR	95% CI	P-value
Work characteristics (vs unemployed)						
Professional/administrative/managerial workers	0.56	0.43–0.75	<0.001***	0.95	0.76–1.18	0.657
Technicians/clerks	0.65	0.48–0.90	0.008**	0.92	0.73–1.17	0.508
Service/sale workers	0.59	0.45–0.78	<0.001***	1.01	0.81–1.25	0.947
Skilled/semi-skilled/unskilled workers	0.63	0.48–0.81	<0.001***	1.08	0.87–1.33	0.482

Adjusted for gender, age group, education, and wealth index quintiles.

AOR = adjusted odds ratio; 95% CI = 95% confidence interval.

*P < 0.05

**P < 0.01

***P < 0.001 (Statistical significance).

### NCD behavioral risk factors by work characteristics and their associations

Among all work characteristics, skilled or semi-skilled, or unskilled workers (43.8%) had the highest proportion of those who were current drinkers, followed by technicians or clerks (34.1%) and service or sale workers (31.2%), respectively (Table 1). Multivariable logistic regression revealed that the likelihood of current drinking was about three times higher among technicians or clerks (95% CI = 2.27–4.10; P-value <0.001) and among skilled, semi-skilled, or unskilled workers (AOR = 2.99; 95% CI = 2.29–3.92; P-value <0.001) compared to the unemployed group. Similar patterns were observed in other work characteristic groups including, service or sale workers (AOR = 2.40; 95% CI = 1.81–3.19; P-value <0.001), and professional or administrative and managerial workers (AOR = 2.85; 95% CI = 1.39–2.48; P-value <0.001) (Table 3).

**Table 3:** Multivariate analysis between work characteristics and behavior risk factors.

Work characteristics	Current drinking (n = 4,925)			Current smoking (n = 4,820)			High dietary risk (n = 4,874)			Insufficient fruit & vegetable intake (n = 4,902)			Insufficient physical activity (n = 2,395)			Sedentary behavior (n = 2,395)		
	AOR	95%CI	P-value	AOR	95%CI	P-value	AOR	95%CI	P-value	AOR	95%CI	P-value	AOR	95%CI	P-value	AOR	95%CI	P-value
**Work characteristics (vs unemployed)**																		
Professional/administrative/managerial workers	1.85	1.39–2.48	<0.001***	1.42	0.96–2.10	0.077***	1.74	1.41–2.13	<0.001***	0.98	0.77–1.25	0.884	1.21	0.88–1.66	0.245	1.05	0.72–1.54	0.785
Technicians/clerks	3.05	2.27–4.10	<0.001***	1.77	1.20–2.62	0.004**	1.89	1.51–2.37	<0.001***	0.91	0.71–1.18	0.489	1.25	0.89–1.76	0.204	0.89	0.60–1.32	0.552
Service/sale workers	2.40	1.81–3.19	<0.001***	1.87	1.29–2.71	0.001**	1.63	1.33–2.01	<0.001***	0.96	0.76–1.22	0.744	1.10	0.80–1.51	0.560	0.82	0.58–1.17	0.281
Skilled/semi-skilled/unskilled workers	2.99	2.29–3.92	<0.001***	2.10	1.49–2.97	<0.001***	1.77	1.45–2.15	<0.001***	0.82	0.65–1.02	0.080**	1.06	0.78–1.44	0.715	0.76	0.54–1.07	0.112

Adjusted for gender, age group, education, and wealth index quintiles.

AOR = adjusted odds ratio; 95% CI = 95% confidence interval.

*P < 0.05

**P < 0.01

***P < 0.001 (Statistical significance).

Additionally, in terms of current smoking among various work characteristics groups, skilled or semi-skilled workers had the highest proportion of current smokers at 30.9%, followed by service or sale workers at 19.8%, and technicians or clerks at 15.7%. The multivariate analysis revealed a two-fold higher likelihood of smoking among skilled, semi-skilled, or unskilled workers compared to the unemployed (95% CI = 1.29–2.71; P-value <0.001). Service or sale workers also showed an increased likelihood, with an adjusted odds ratio of 1.87 (95% CI = 1.29–2.71; P-value = 0.001), followed by technicians or clerks with an AOR of 1.77 (95% CI = 1.20–2.62; P-value = 0.004), and professional or administrative and managerial workers (AOR = 1.42; 95% CI = 0.96–2.10; P-value = 0.077) (Table 3).

More than half of samples in all work characteristics reported having high dietary risks accounting for 67.9% of technicians or clerks, 66.7% of professional or administrative and managerial workers, 66.5% of skilled or semi-skilled, or unskilled workers, and 64.1% of service or sale workers. Furthermore, 22.0 to 32.2% of all work categories reported insufficient consumption of vegetables and fruits, with skilled or semi-skilled and unskilled workers having the highest insufficient proportion among the four groups (Table 1). The multivariate analysis revealed around two times higher chance of having high dietary risks among technicians or clerks compared to the unemployed (AOR = 1.89; 95% CI = 1.51–2.37; P-value <0.001). Similarly, skilled or semi-skilled, or unskilled workers (AOR = 1.77; 95% CI = 1.45–2.15; P-value <0.001), professional or administrative and managerial workers (AOR = 1.74; 95% CI = 1.41–2.13; P-value <0.001), and service or sale workers (AOR = 1.63; 95% CI = 1.33–2.01; P-value <0.001) also tend to have high dietary risks. Interestingly, the variable related to inappropriate fruit and vegetable intake did not show statistically significant association with work characteristics (Table 3).

About 26.2 to 31.4% of samples reported having insufficient physical activity and about 75.4 to 83.4% had a sedentary lifestyle. Notably, professional administrative or managerial workers had the highest levels of both insufficient physical activity and sedentary behavior compared to the other groups accounting for 31.3 and 79.9%, respectively (Table 1). However, the multivariate analysis did not reveal significant associations between insufficient physical activity and sedentary behavior and any of the types of work characteristics (Table 3).

## Discussion

Based on our understanding, this study is one of the studies focusing on the correlation between work characteristics and non-communicable diseases (NCDs), overweight/obesity, and behavioral risk factors. The main finding of this study indicates that all or nearly all work characteristics are associated with NCDs, current drinking, current smoking, and high dietary risk. However, no association is observed with overweight or obesity, insufficient fruit and vegetable intake, insufficient physical activity, and sedentary behavior. Nevertheless, the discussion regarding the study findings is divided into four main sections.

Firstly, this study suggests that all work characteristics are associated with NCDs, but no association is observed with overweight or obesity. Several studies have highlighted that NCDs result from multiple factors, rather than one direct cause and it is not a causal relationship with obesity. These factors beyond work environment include growing urbanization, changing lifestyles, population growth, and epidemiologic shifts in diseases, as well as sociocultural and environmental influences such as changes in air quality [25–27]. When considering each work characteristics group in this study, it is found that the unemployed group has the highest proportion of NCDs compared to other work characteristics. It is similar to other studies that NCDs are more likely to be found in unemployed [28]. NCDs has an effect on working productivity leading to unemployment and the unemployed person are more like to have self-report poor health compared to the employed ones [29].

Furthermore, it was found that the work characteristics in Bangkok has a lower likelihood of NCDs, while the other risk factors remain high compared to the unemployed, can be attributed to several factors. A key factor is the enhanced access to health screening programs available to those who are employed. Regular screenings facilitate early detection and management of conditions such as hypertension, diabetes, and cardiovascular diseases, which are significant contributors to reduction of NCDs [30]. Workplace health initiatives and insurance coverage often support these services, increasing health awareness and encouraging healthier lifestyle choices [31]. Research also indicates that health screenings lead to improved outcomes, including lower blood pressure and higher diagnosis rates, thereby reducing the long-term risk of NCDs [32]. This is particularly relevant in urban areas like Bangkok, where employed individuals generally have greater access to structured healthcare services than the unemployed, who may have fewer opportunities for preventive care.

Secondly, the results of this study that all work characteristics were significantly with alcohol and smoking behaviors and having a high dietary risk especially in skilled/semi-skilled/unskilled workers. A previous study conducted in Bangkok found that skilled or semi-skilled workers, such as construction workers, had a problem drinking rate of 37.5% and an alcohol dependence rate of 36.7% [33]. In the survey of health behavior of employees in an enterprise, it was found that manual workers have higher drinking and smoking habits than non-manual workers [34]. Similar findings were observed in a study conducted in India, where unskilled workers were identified as a marginalized sector of the community. This is attributed to their lower socio-economic status, lack of health awareness, and high mechanical workload, which contribute to unhealthy lifestyle behaviors [35].

Meanwhile, technicians or clerks, as well as service or sales workers, were also correlated with behaviors such as drinking, smoking, and having a high dietary risk, potentially influenced by stress. Work-related stress is related to work contents such as job content (monotony, under-stimulation, lack of variety), work load and work pace (too much or too little to do, work under time pressure), working hours (strict or inflexible, long and unsocial, unpredictable, badly designed shift systems), and participation and control (lack of participation in decision-making, lack of control over work processes, pace, hours, methods, and the work environment) [36]. These working conditions are commonly found in tasks of technicians, clerks, service or sales workers. For instance, occupational hazards can induce stress, exacerbating the overall impact on health [37]. Example in Thailand studies have revealed that technicians of maintenance and repair department in hospital face health risks associated with alcohol consumption, smoking, and being overweight [38]. Similar findings were observed in a study conducted in Japan, which examined the relationship between job (office clerk, planner, sales, engineer, industrial technician, industrial worker) stress factors and behavioral risk factors [39]. Thus, stress in workplace should be focused to prevent effect to health outcome especially in NCDs.

Thirdly, although this study found that all group of workers did not significantly exhibit insufficient physical activity and sedentary behavior. However, professional administrative, or managerial workers, who belong to the high-income group and have completed at least a diploma or higher education level, exhibited a higher proportion of insufficient physical activity and higher levels of sedentary behavior compared to another group of workers, which is similar the findings study from Australia and China [40–42]. In Thailand, the prevalence of low physical activity/high sedentary behavior among office-based workers is 48.3%, compared to 26.9% of unemployed and 24.8% of labor-intensive workers. The differences arise because those engaged in labor-intensive work involve a lot of body movement, whereas office workers engage in minimal physical activity and sitting time for extended periods of time. Moreover, Topothai’s study explained that there is a transition in the labor market in Thailand from physically demanding jobs to more sedentary ones aligning with global and regional situations, especially in lower- and middle-income countries [43]. Therefore, building a workplace environment can lead to increased physical activity and reduced sitting time for work such as time of day, distance to activity, work planning, using sit‐stand desks, walking during breaks or length of breaks [44,45].

Finally, the results indicated that all work characteristics were significantly associated with high dietary risk, although there is no difference among each group of workers, but the food environment in the workplace may be a problem, and the opportunities the environment provides to purchase both healthy and unhealthy food are important influences on food choices [46]. Several studies show that food environments nearby workplaces are associated with certain eating behaviors, for example, the distance and density of food outlets around the workplace, and access to healthy foods in the workplace is often limited compared to an abundance of unhealthy foods present in workplace canteens, onsite shops, and vending machines [47–50]. Therefore, substantial resources have been invested in attempts to both improve healthy food access and reduce unhealthy food sources within the workplace, such as providing healthy food options (such as vegetables and fruit) and increasing choice in the canteen for employees [51,52].

However, it is important to acknowledge certain limitations of this study and consider potential solutions. Firstly, the reliance on cross-sectional data restricts our ability to track changes over time or establish long-term cohort effects especially in NCD development. Secondly, the use of self-report measures introduces the potential for recall bias and inaccuracies specifically in BMI, behavioral risk factors and NCD status report. Thirdly, it should be noted that mental health conditions were not included in the scope of non-communicable diseases in this study while it is recognized as a part of NCDs. To address study limitations, future research should adopt longitudinal designs and include objective measures like medical records. The scope of workplace NCD studies should encompass mental health and consider additional factors such as working hours and environment, as well as socioeconomic status.

Despite limitations, this study is a pioneering investigation into NCDs and occupation in Bangkok’s urban context, utilizing national survey data. It highlights the importance of NCD interventions in workplaces and diverse interventions targeting different working conditions especially for informal sector. Advocacy for healthier environments in workplaces, including tobacco-free and wellness programs in workplaces, is supported by international organizations [53–55]. In Thailand, NCD prevention in the workplace is guided by Ministry of Public Health and Ministry of Labour guidelines, although not mandated by law [56]. Therefore, key recommendations adjusted to Thai context are as follows. Firstly, obesogenic environments, particularly in the workplace, significantly contribute to non-communicable diseases (NCDs) [57]. While international strategies often target individual risk factors, promoting a healthier workplace requires universal application of setting based NCD policies, such as smoking regulations, healthy canteens and promoting opportunities for physical activity, integrated into broader regulatory frameworks for effective outcomes [58,59]. Secondly, utilizing NCD-related workplace data, such as national surveys, is crucial for informing targeted health policies. For instance, policies addressing smoking, tobacco, and unhealthy eating should focus on vulnerable groups like blue-collar workers, the poor, or those with low literacy. Thirdly, beyond regulations, incentives whether financial (such as tax credits) or capacity-building-related interventions (access to government support), can be instrumental in motivating businesses to implement workplace NCD interventions. Next, we only focused on the variables that are available from the survey; there are other potential factors that should be considered in the future, such as access to health care services. Finally, workplaces are urged to prioritize mental well-being within the 5x5 Non-Communicable Disease (NCD) agenda, recognizing its influence on physical health and its link to risk behaviors. Specifically in Thailand, there is a call to integrate mental health prevention into labor laws, emphasizing the need to address stress through organizational measures and improve working and living conditions [60,61].

## Conclusion

The study revealed a relationship between work characteristics and NCDs. Employees exhibited a decreased probability of reporting non-communicable diseases (NCDs) in contrast to those who were not employed. All working group demonstrated a notable correlation with alcohol consumption, smoking, and a heightened risk of dietary intake, specifically in individuals in skilled, semi-skilled, or unskilled occupations, as well as technicians or clerks and service or sales workers. Accordingly, the working environment seem to affect health status due to repetition and stress in workplace leading to alcohol drinking and tobacco use. Accordingly, the working environment seem to affect health status due to repetition and stress in workplace leading to alcohol drinking and tobacco use including high dietary risks. As a result, it is imperative to promote healthier workplaces, such as smoking regulations, healthy canteens, and promoting opportunities for physical activity, which is essential under the labour law. Additionally, utilizing NCD-related workplace data including mental well-being and implementing incentives can motivate businesses to prioritize NCD interventions. Future research should employ longitudinal study designs and incorporate objective measures, such as medical records. The scope of workplace studies on non-communicable diseases should be expanded to include mental health and consider additional factors, including working hours, workplace environment, and socioeconomic status.
